# Safety of Sabin Inactivated Poliovirus Vaccine Administered Standalone or Concomitantly with Other Childhood Vaccines: A Real-World Study in China

**DOI:** 10.3390/vaccines14020161

**Published:** 2026-02-09

**Authors:** Binbing Wang, Fanya Meng, Wenqing Xue, Ying Su, Tingyi Jiang, Yan Dong, Mingxue Ren, Jihai Tang

**Affiliations:** Department of Immunization Program, Anhui Provincial Center for Disease Control and Prevention, Hefei 230601, China; wbb@ahcdc.com.cn (B.W.); mfy@ahcdc.com.cn (F.M.); xuewenqin@ahcdc.com.cn (W.X.); sy@ahcdc.com.cn (Y.S.); jty@ahcdc.com.cn (T.J.); dy@ahcdc.com.cn (Y.D.); rmx@ahcdc.com.cn (M.R.)

**Keywords:** Sabin inactivated poliovirus vaccine, adverse events following immunization, concomitant vaccination, vaccine safety, real-world study, China

## Abstract

**Background/Objectives**: Sabin strain-based inactivated poliovirus vaccine (sIPV) is increasingly used in China’s routine immunization program and is often administered concomitantly with other childhood vaccines. However, large-scale real-world evidence on the safety of concomitant sIPV vaccination remains limited. This study evaluated the safety of sIPV administered standalone or concomitantly with other routine vaccines using provincial surveillance data. **Methods**: A retrospective observational study was conducted using data from the China National Adverse Events Following Immunization Surveillance System and the Anhui Provincial Immunization Information Management System. All sIPV doses administered between 1 November 2023 and 31 July 2025 were included. AEFI reporting rates per 100,000 doses were calculated. Descriptive analyses were performed by demographic characteristics and dose number. Multivariable negative binomial regression models were used to assess the association between vaccination mode and AEFI incidence, with dose-stratified analyses when appropriate. **Results**: Among the 303,526 sIPV doses analyzed (135,550 standalone and 167,976 concomitant), 188 AEFI cases were reported, yielding an overall reporting rate of 61.94 per 100,000 doses. Most AEFI were mild, self-limited general reactions, mainly fever and local injection-site reactions. Only two serious AEFI were reported, both resolving without sequelae. After adjustment for confounders, no significant difference in overall AEFI incidence was observed between standalone and concomitant vaccination (aRR = 0.97, 95% CI: 0.64–1.47). AEFI reporting was associated with age and region, while no consistent dose-related trend was identified. **Conclusions**: sIPV showed a favorable safety profile when administered standalone or concomitantly with other routine vaccines in real-world settings. Concomitant vaccination did not increase AEFI risk, supporting the continued use of sIPV in routine immunization programs.

## 1. Introduction

Poliomyelitis is an acute infectious disease caused by poliovirus that primarily affects children and may result in irreversible paralysis or even death. Since the launch of the Global Polio Eradication Initiative (GPEI), global poliomyelitis incidence has declined dramatically as a result of sustained high vaccination coverage worldwide [1,2]. However, in the final stage of global polio eradication, how to maintain population immunity while minimizing vaccine-associated risks remains a critical public health challenge for national immunization programs [3].

Historically, oral poliovirus vaccine (OPV) has played a pivotal role in polio control and elimination. Nevertheless, the risks associated with OPV, including vaccine-associated paralytic poliomyelitis (VAPP) and vaccine-derived polioviruses (VDPVs), have led to a global consensus on the gradual transition from OPV to inactivated poliovirus vaccine (IPV) [4,5]. As IPV contains no live virus, it offers a higher level of biosafety and has become an essential component of current polio immunization strategies [6].

sIPV is produced using attenuated Sabin strains as antigens. Compared with conventional Salk strain-based IPV derived from wild polioviruses, sIPV has advantages in terms of production safety and biosafety requirements [7]. In recent years, sIPV has been evaluated through multiple clinical trials and real-world studies and has been progressively incorporated into routine immunization programs [8,9]. In China, an sIPV developed by Beijing Sinovac Biotech Co., Ltd., a subsidiary of Sinovac Biotech Ltd., was approved for marketing by the National Medical Products Administration on 12 July 2021, and subsequently obtained World Health Organization (WHO) prequalification in 2022 [10,11]. Since then, sIPV has been widely used in both routine immunization and supplementary vaccination activities.

In routine immunization practice, concomitant administration of vaccines is commonly adopted to improve service efficiency, reduce clinic visits, and enhance caregiver compliance [12]. According to China’s current and updated immunization schedules, sIPV is scheduled to be administered concomitantly with diphtheria–tetanus–acellular pertussis vaccine (DTaP) at specific ages, and may also be co-administered on the same day with other vaccines, such as Haemophilus influenzae type b (Hib) vaccine and rotavirus vaccine [13]. Although concomitant vaccination is programmatically necessary, concerns remain among the public and healthcare providers regarding whether co-administration may increase the risk of adverse events following immunization (AEFI), particularly during the early phase of introduction of newly licensed vaccines or new vaccination strategies [14].

At present, evidence regarding the safety of Sabin strain–based inactivated poliovirus vaccine (sIPV) is derived mainly from clinical studies and a limited number of post-marketing surveillance reports [9,15,16]. Clinical trials are typically constrained by relatively small sample sizes and therefore may not fully capture the occurrence of adverse events across different dose numbers, diverse population characteristics, or multiple concomitant vaccination combinations [17]. Although previous studies have consistently suggested a favorable safety profile for sIPV, systematic safety evaluations focusing on concomitant administration of sIPV with other vaccines remain insufficient, particularly in large-scale, real-world populations, where evidence is still limited [16].

The China National Adverse Events Following Immunization Surveillance System (CNAEFIS), a nationwide passive surveillance platform, provides an important data source for conducting post-marketing real-world vaccine safety studies [18]. Analyses based on this system enable evaluation of vaccine safety under routine programmatic conditions from a public health perspective and can inform optimization of immunization strategies [19,20].

Against this background, the present study utilized CNAEFIS data from Anhui Province to systematically assess adverse events following immunization (AEFI) associated with sIPV administered standalone or concomitantly with other vaccines between 1 November 2023 and 31 July 2025. By integrating descriptive analyses with multivariable regression models, we examined whether concomitant vaccination was associated with changes in AEFI reporting rates and further explored safety profiles across different population subgroups and dose numbers. This study aims to provide real-world evidence to support concomitant use of sIPV within China’s immunization program and to inform the rational application of sIPV in the context of global polio eradication.

## 2. Materials and Methods

### 2.1. Study Design and Population

The study population comprised individuals in Anhui Province who received Sinovac sIPV in accordance with the routine immunization schedule, either administered standalone or concomitantly with other vaccines, between 1 November 2023 and 31 July 2025. In addition, a separate subgroup analysis was conducted among children who were born between 1 March 2016 and 30 September 2019 and who had received only one dose of IPV (including IPV-containing combination vaccines) or had no prior history of IPV vaccination. These children received a second dose of IPV as part of a catch-up immunization program between 16 January 2024 and 30 June 2024.

All vaccinations were administered in accordance with the Vaccination Work Specifications. Informed consent was obtained prior to vaccination. Before administration, vaccination staff informed the guardians of vaccine recipients about the vaccine type, purpose, relevant contraindications, and potential adverse reactions and conducted a health status assessment. Vaccination was performed by qualified healthcare personnel for individuals without contraindications.

All vaccinations were administered in accordance with national immunization regulations. Informed consent was obtained from the guardians of vaccine recipients at the time of vaccination as part of routine immunization services.

This study involved the secondary analysis of de-identified data collected through routine AEFI surveillance systems. As no identifiable personal information was used and no additional interventions were performed, ethical review and study-specific informed consent were waived.

### 2.2. Vaccines and Immunization Schedule

The Sabin strain–based inactivated poliovirus vaccine (Vero cell–derived) was used in this study. Each dose (0.5 mL) was administered via intramuscular injection and contained inactivated poliovirus of type 1, 2, and 3 at 15, 45, and 45 D-antigen units, respectively. All vaccine batches were released after quality testing and certification by the National Institutes for Food and Drug Control of China.

According to the National Immunization Schedule for Children (2021 edition), the poliomyelitis vaccination schedule in China consists of two doses of IPV administered at 2 and 3 months of age, followed by two doses of oral poliovirus vaccine (OPV) administered at 4 months of age and at 4 years of age. The IPV doses at 3 and 4 months of age are administered concomitantly with diphtheria–tetanus–acellular pertussis vaccine (DTaP), whereas the doses at 2 months of age and 4 years of age are administered standalone. In addition, in routine practice, some non–national immunization program vaccines may also be administered concomitantly with sIPV.

In 2024, the National Disease Control and Prevention Administration of China issued the Notice on Adjustments to the National Immunization Schedule for DTaP and DT Vaccines, announcing that, effective 1 January 2025, the existing immunization schedule of administering one dose of DTaP at 3, 4, 5, and 18 months of age and one dose of diphtheria–tetanus (DT) vaccine at 6 years of age would be adjusted nationwide. Under the revised schedule, one dose of DTaP vaccine is to be administered at 2, 4, 6, and 18 months of age, as well as at 6 years of age.

Vaccination mode was categorized as: (1) standalone vaccination, defined as administration of sIPV alone; and (2) concomitant vaccination, defined as administration of sIPV on the same day with at least one other vaccine.

Furthermore, in response to the national catch-up immunization program, Anhui Province implemented a supplementary vaccination campaign in 2024 targeting children born between 1 March 2016, and 30 September 2019, who had received only one dose of IPV (including IPV-containing combination vaccines) or had no prior history of IPV vaccination. These children were administered a second dose of IPV as part of the catch-up strategy.

Vaccines administered concomitantly with sIPV, other than DTaP, were determined according to the types of vaccines actually used in Anhui Province during the study period.

### 2.3. Vaccination Data

Vaccination data were obtained from the Anhui Provincial Immunization Information Management System. The dataset included the number of administered doses between 1 November 2023 and 31 July 2025, covering sIPV administered standalone, DTaP administered standalone, other vaccines eligible for concomitant administration with sIPV administered standalone, as well as doses of sIPV administered concomitantly with other vaccines.

### 2.4. Safety Data

Safety surveillance data were obtained from the CNAEFIS, a nationwide passive surveillance platform for post-marketing vaccine safety monitoring [18]. Reporting, investigation, and case management (including causality assessment) were conducted in accordance with the National Surveillance Program for Suspected Adverse Events Following Immunization [21], and causality assessment followed the criteria recommended by the WHO [22].

For this analysis, individual case reports of adverse events following immunization (AEFI) occurring between 1 November 2023 and 31 July 2025 were collected for sIPV administered standalone, vaccines eligible for concomitant administration with sIPV administered standalone, and sIPV administered concomitantly with other vaccines.

Except for routine post-vaccination reactions such as fever (axillary temperature), local erythema, swelling, and injection site induration, all reported AEFI cases were subject to investigation by an expert AEFI investigation and diagnostic panel. Once an event was confirmed as an AEFI, it was classified into one of five categories according to the underlying cause: adverse reactions (including common reactions and rare or abnormal reactions), vaccine quality defects, immunization errors, coincidental events, and psychogenic reactions. The present study primarily focused on adverse reactions, defined as unintended and harmful responses that occur following administration of a qualified vaccine under standard vaccination practices, including both common and abnormal reactions. In addition, the occurrence of coincidental events was also assessed.

Based on severity, AEFI were further categorized as serious AEFI or non-serious AEFI.

### 2.5. Statistical Analysis

AEFI case reports associated with sIPV administered standalone or concomitantly with other vaccines were extracted and compiled from the immunization information systems. Clinical diagnoses and classifications of adverse reactions for all reported cases were reviewed by AEFI surveillance professionals at the Anhui Provincial Center for Disease Control and Prevention. Vaccination records were then matched with corresponding immunization data from the Immunization Information Management System, followed by data anonymization. A study database was established using Microsoft Excel, and statistical analyses were performed using SAS software (version 9.4).

A retrospective analysis was conducted to assess the occurrence of AEFI following sIPV administered standalone or concomitantly with other vaccines. Descriptive analyses included AEFI types, composition proportions, and distributions by demographic characteristics (age and sex), calendar time, dose number, and geographic region. The AEFI reporting rate was defined as the number of reported AEFI events divided by the number of vaccine doses administered, multiplied by 100,000 doses. The composition proportion of AEFI was defined as the number of events of a specific AEFI category divided by the total number of AEFI events, multiplied by 100%. As this study was based on a passive AEFI surveillance system, no predefined post-vaccination risk window was applied as an inclusion criterion. All reported AEFI linked to sIPV doses during the study period were included. Time-to-onset intervals were summarized descriptively to characterize the temporal distribution of reported events.

For crude comparisons, relative risks (RRs) and corresponding 95% confidence intervals (95% CIs) were calculated to describe differences in AEFI reporting rates between standalone vaccination and concomitant vaccination. Comparisons of rates across groups were primarily descriptive, with inferential conclusions based mainly on regression model results. Comparisons between two or multiple groups were conducted using the chi-square test; when expected cell counts were less than 1 or sample sizes were small, Fisher’s exact test was applied. For multiple comparisons, Bonferroni correction was used, with the adjusted significance level set at α’ = 0.05/k. These tests were primarily used to describe distributional differences across subgroups.

Given that AEFI events represent low-frequency count outcomes and that the number of administered doses served as the exposure, Poisson regression models were initially applied to model AEFI incidence rates. In the Poisson regression model, substantial overdispersion was detected (deviance/df = 2.28; Pearson χ^2^/df = 24.47). Therefore, negative binomial (NB) regression models were used as the primary analytical approach to obtain robust parameter estimates and standard errors. In multivariable models, covariates included vaccination mode (standalone vs. concomitant), age in months, sex, geographic region, and dose number, with the number of administered doses included as an offset term. Effect estimates were expressed as adjusted rate ratios (aRRs) with 95% CIs, and statistical significance was evaluated using Type III likelihood ratio tests.

Considering potential heterogeneity across dose numbers due to differences in immunization schedules, an interaction term between vaccination mode and dose number was further incorporated into the models. When significant interaction effects were identified, stratified analyses by dose number were conducted. All statistical tests were two-sided, and a significance level of α = 0.05 was applied.

For geographic analyses, assuming a uniform AEFI reporting rate across the province, the expected number of AEFI cases in each region was calculated based on the number of administered doses. Standardized residuals were then used to describe deviations of regional reporting levels from the provincial average. This analysis was intended to characterize regional differences in reporting patterns rather than to compare true underlying risks.

## 3. Results

### 3.1. Baseline Characteristics

#### 3.1.1. Demographic Characteristics

During the study period (1 November 2023 to 31 July 2025), a total of 303,526 vaccine doses were included in the analysis, of which 135,550 doses (44.7%) were administered as sIPV standalone and 167,976 doses (55.3%) were administered concomitantly with other vaccines.

The mean age of vaccine recipients was 11.7 ± 21.85 months. Recipients in the standalone sIPV group had a mean age of 12.8 ± 24.00 months, whereas those in the concomitant vaccination group had a mean age of 10.7 ± 19.90 months; this difference was statistically significant (*p* < 0.0001). The overall median age was 3 months, with a range of 1–746 months.

Age distribution analysis showed that the majority of vaccinations were administered to infants aged 2 months (33.37%) and those aged 3 months (31.11%), followed by infants aged 4–17 months (16.90%) and children aged 18 months–<4 years (7.60%). Individuals aged ≥18 years accounted for only 0.01% of the vaccinated population. The distribution across age groups differed significantly (*p* < 0.0001).

Regarding sex distribution, 161,970 recipients were male (53.36%) and141,556 were female (46.64%), with no significant difference between groups (*p* = 0.0056).

Geographic distribution indicated that vaccine recipients were primarily concentrated in Fuyang City (20.44%), Hefei City (16.82%), Chuzhou City (12.95%), and Bozhou City (8.46%). Significant differences were observed across regions (*p* < 0.0001), with Fuyang City accounting for the largest proportion of administered doses (Table 1).

#### 3.1.2. Vaccines Administered Concomitantly

As shown in Supplementary Table S1, among the 167,976 doses of sIPV administered concomitantly during the study period, a wide variety of vaccines were co-administered, comprising a total of 199,903 concomitant vaccine doses.

The most frequently co-administered vaccine with sIPV was diphtheria–tetanus–acellular pertussis vaccine (DTaP), with 117,878 doses, accounting for 58.97% of all concomitant vaccinations. This was followed by the pentavalent rotavirus vaccine (19,525 doses, 9.77%) and Haemophilus influenzae type b (Hib) vaccine (13,583 doses, 6.79%). Other vaccines commonly co-administered with sIPV included the DTaP–Hib quadrivalent vaccine (11,079 doses, 5.54%), hepatitis B vaccine (9332 doses, 4.67%), varicella vaccine (6872 doses, 3.44%), and diphtheria–tetanus (DT) vaccine (4338 doses, 2.17%).

Additional vaccines administered concomitantly with sIPV included measles–mumps–rubella (MMR) vaccine (3903 doses, 1.95%), influenza vaccine (2143 doses, 1.07%), rotavirus vaccine (1925 doses, 0.96%), hepatitis A vaccine (1564 doses, 0.77%), Japanese encephalitis vaccine (1384 doses, 0.69%), bacille Calmette–Guérin (BCG) vaccine (165 doses, 0.08%), and enterovirus 71 (EV71) vaccine (135 doses, 0.07%).

Overall, sIPV was most commonly co-administered with DTaP, Hib, and rotavirus vaccines, which together accounted for approximately 75.7% of all concomitant vaccination doses. The proportions of other vaccines administered concomitantly with sIPV were each below 0.05% (Supplement Table S1).

Among AEFI reported following concomitant administration with sIPV, most were associated with vaccines containing DTaP components. Acellular pertussis–containing vaccines (DTaP) accounted for 80 cases (73.39%), predominantly mild general reactions, while the DTaP–Hib combination vaccine accounted for 7 cases (6.42%). Together, these vaccines comprised 79.82% of all reported AEFI following concomitant administration (Supplementary Table S2).

### 3.2. AEFI Outcomes

#### 3.2.1. Overall Occurrence of AEFI

During the study period, a total of 188 AEFI cases were reported, corresponding to an overall reporting rate of 61.94 per 100,000 administered doses.

The AEFI reporting rates were 58.28 per 100,000 doses in the standalone sIPV group and 64.89 per 100,000 doses in the concomitant vaccination group. Based on unadjusted comparisons of reporting rates, no statistically significant difference was observed between the two groups (*p* = 0.4669).

In addition, the overall AEFI reporting rate for standalone administration of DTaP and DTaP-containing combination vaccines was calculated separately and found to be 89.78 per 100,000 doses. A statistically significant difference in AEFI reporting rates was observed among the three groups—standalone sIPV, concomitant vaccination, and standalone DTaP/combination vaccines (*p* < 0.0001).

Most AEFI cases occurred within three days after vaccination, with the highest proportion reported within 30 min post-vaccination. The majority of reported AEFI were general reactions (97.87%), whereas reports of abnormal reactions and coincidental events were rare. During the study period, two serious AEFI cases were reported, both occurring in the concomitant vaccination group; however, the incidence was extremely low, and no statistically significant difference was observed between groups (*p* = 0.5057).

All reported AEFI cases resulted in recovery or improvement, with no deaths or serious sequelae reported. These findings indicate that both standalone and concomitant administration of sIPV were associated with a favorable overall safety profile, with no evidence of a significant increase in risk (Table 2).

Geographic analysis showed that AEFI reporting rates varied across different prefecture-level cities. The number of AEFI cases reported in Fuyang City was higher than the expected value calculated based on the provincial average reporting rate, with a positive standardized residual, whereas several other cities reported fewer cases than expected. These findings suggest heterogeneity in AEFI reporting levels across regions.

It should be emphasized that this analysis was based on data from a passive surveillance system and therefore reflects regional differences in AEFI reporting rather than direct comparisons of true underlying risk. The observed differences may be influenced by factors such as variations in vaccination volume, surveillance system sensitivity, and reporting practices across regions (Table 3).

#### 3.2.2. Between-Group Comparison of AEFI Risk

The relative risks of different types of AEFI were generally comparable between the standalone sIPV group and the concomitant vaccination group.

The relative risk for general reactions was 0.91 (95% CI: 0.68–1.22), and the relative risk for abnormal reactions was 1.24 (95% CI: 0.08–19.81). As the confidence intervals for both estimates included the null value, no statistically significant differences were observed between the two groups.

Given the low overall incidence of AEFI and the lack of adjustment for differences in population characteristics in crude rate comparisons, these findings are primarily descriptive. Further risk assessment was therefore based on the results of multivariable regression analyses.

#### 3.2.3. Distribution of AEFI by Age, Sex, and Region

Stratified by age, AEFI were mainly reported among infants aged 2 months and those aged 3 months. In the 2-month age group, the AEFI reporting rate was higher in the standalone vaccination group than in the concomitant vaccination group (28.77 vs. 17.26 per 100,000 doses; *p* = 0.0352). While among children aged 4–17 months, the rate was lower in the standalone vaccination group (6.64 vs. 17.26 per 100,000 doses; *p* = 0.0093). No statistically significant differences were observed in the remaining age groups (all *p* > 0.05).

With respect to sex, no statistically significant difference in AEFI reporting rates was observed (*p* > 0.05).

Regarding geographic distribution, Fuyang City reported the highest number of AEFI cases. Overall, AEFI reporting rates were comparable across regions and between vaccination strategies, with the exception of Huaibei, where a statistically significant difference was observed between the standalone and concomitant vaccination groups (*p* = 0.0367) (Table 4).

#### 3.2.4. Multivariable Regression Analysis

Multivariable regression analyses indicated substantial overdispersion in the Poisson regression model. After adopting a negative binomial regression model, model fit improved markedly (deviance/df = 2.00, Pearson χ^2^/df = 21.01). 

In the NB regression model, age in months was associated with AEFI reporting (*p* = 0.0126), whereas sex, dose number, and vaccination mode were not significant predictors. Regional differences in AEFI reporting were observed (*p* = 0.0355), which may reflect heterogeneity in reporting practices. No statistically significant association was observed between vaccination mode and AEFI reporting rates (aRR = 0.97, 95% CI: 0.64–1.47; *p* = 0.8860), indicating that concomitant vaccination did not significantly alter the AEFI reporting rate.

When an interaction term between vaccination mode and dose number was introduced, a statistically significant interaction was observed (*p* = 0.0366), indicating that the association between vaccination mode and AEFI reporting varied across dose numbers. Age in months and region remained independently associated with AEFI reporting.

#### 3.2.5. Distribution of AEFI by Dose Number

In dose-specific NB regression analyses, no statistically significant differences in AEFI reporting rates were observed between concomitant and standalone vaccination across the first, second, and fourth doses. Although point estimates varied across doses, confidence intervals were wide and consistently crossed unity, indicating no evidence of increased risk associated with concomitant vaccination. These findings should be interpreted cautiously due to sparse data in certain strata and the descriptive nature of subgroup analyses.

For doses 3–5, AEFI incidence rates were all below 2.0 per 100,000 doses, with no significant between-group differences (all *p* > 0.05).

AEFI cases were predominantly observed after the first and second doses, accounting for more than 85% of all reports. A Cochran–Armitage trend test suggested a non-significant decreasing tendency in AEFI occurrence with increasing dose number, although this trend did not reach statistical significance in the two-sided test (Z = –1.76, two-sided *p* = 0.078) (Table 5).

### 3.3. Main Diagnoses of AEFI

#### 3.3.1. AEFI by Primary Diagnosis

As shown in Table 6, general reactions constituted the vast majority of AEFI, accounting for 97.2% of all reported events, with an overall incidence rate of 60.62 per 100,000 doses. Fever was the most common manifestation (38.88 per 100,000 doses). Most cases were mild to moderate in severity (37.6–38.5 °C). This was followed by injection-site erythema/swelling (18.12 per 100,000 doses) and injection-site induration (14.50 per 100,000 doses). The incidence of rash allergic was 1.98 per 100,000 doses, with no significant difference between groups (*p* = 1.0000). 

Abnormal reactions (0.66 per 100,000 doses), thrombocytopenic purpura (0.33 per 100,000 doses), and coincidental events (0.33 per 100,000 doses) were all extremely rare.

A total of two serious AEFI cases were reported, both occurring in the concomitant vaccination group and diagnosed as thrombocytopenic purpura (Table 6). Two serious AEFI cases were identified during the study period, both following concomitant vaccination. In one case (sIPV + DTaP–Hib), a causal association with vaccination could not be ruled out, whereas in the other case (sIPV + DTaP), the causality assessment was undetermined (Supplementary Table S3).

#### 3.3.2. Age-Specific Distribution of Primary AEFI Diagnoses

Across all age groups, AEFI incidence rates were low and predominantly consisted of general reaction symptoms. No statistically significant differences in AEFI incidence were observed between separate and concomitant vaccination within any age group (all *p* > 0.05). Event counts were sparse in several strata, particularly among older age groups, and results should therefore be interpreted cautiously (Supplementary Table S4).

### 3.4. Safety Analysis During the Large-Scale Catch-Up Vaccination Campaign

A preliminary safety analysis was conducted among individuals born between 1 March 2016 and 30 September 2019. Only two general reaction cases were reported in the concomitant vaccination group, corresponding to an incidence rate of 7.61 per 100,000 doses. No AEFI were reported among children aged 5–8 years during the large-scale catch-up vaccination period (vaccination dates from 30 January to 30 June 2024) within this birth cohort.

## 4. Discussion

Based on AEFI surveillance data from Anhui Province, this study systematically evaluated the safety of sIPV administered standalone and concomitantly with other vaccines between November 2023 and July 2025. Using more than 303,000 doses of real-world vaccination data, and integrating descriptive analyses with multivariable negative binomial regression models, we assessed the overall safety profile of concomitant sIPV vaccination as well as its performance across different populations and dose numbers.

The results showed that the overall reporting rate of AEFI following sIPV vaccination was low and was dominated by mild, self-limited general reactions. No new or unexpected safety signals were identified. Regardless of whether sIPV was administered standalone or concomitantly with other vaccines, the overall incidence of AEFI remained low; serious AEFI were extremely rare and, after investigation, all cases improved or recovered, with no deaths or severe sequelae reported. This overall safety profile is consistent with findings from previous post-marketing surveillance and/or provincial monitoring studies conducted in China [16,23,24,25].

In addition, we summarized AEFI reporting rates for other IPV-containing vaccines administered during the same period for contextual reference. The AEFI reporting rate was 109.75 per 100,000 doses for a pentavalent vaccine containing IPV and 317.97 per 100,000 doses for standalone conventional IPV (cIPV, Pasteur), both of which were higher than that observed for sIPV in the present study (61.94 per 100,000 doses). These cIPV data were not included in the main comparative analyses, as the primary objective of this study was to evaluate the safety of sIPV; however, they provide useful background context for interpreting the favorable safety profile of sIPV observed in real-world use.

After accounting for overdispersion by using NB regression, no significant association between concomitant vaccination and AEFI incidence was identified. These findings are generally consistent with previous post-marketing surveillance studies of inactivated poliovirus vaccines [16,23,24], which have similarly reported that no evidence of increased risk associated with concomitant administration. This interpretation is further supported by the World Health Organization’s assessments of poliovirus vaccine safety in routine immunization programs, which endorse concomitant vaccination practices as safe and programmatically appropriate [26,27].

In this study, sIPV was most frequently co-administered with DTaP, Hib, and rotavirus vaccines, together accounting for approximately three-quarters of all concomitant doses, reflecting the predominant real-world vaccination scenarios in current immunization programs. Previous studies evaluating the concomitant administration of sIPV with DTaP—including clinical trials and post-marketing studies—have similarly reported comparable safety profiles, consistent with our findings that concomitant vaccination does not increase overall risk [9,16,24,28]. According to WHO SAGE, adverse events reported after IPV administered in combination with other vaccines are difficult to differentiate from those caused by the accompanying vaccines (e.g., DTaP) [27].

Most reported AEFI, including the two serious cases identified in this study, were associated with DTaP-containing vaccines, which are known to have higher baseline AEFI reporting rates. Notably, no serious AEFI were reported following standalone sIPV administration. However, given the small number of serious events and the inherent limitations of a passive surveillance system, causal attribution to specific non-polio components cannot be conclusively determined.

The observed regional differences in AEFI reporting are likely attributable to differences in reporting behavior, population composition, or random variation inherent to passive surveillance systems, rather than a true change in risk attributable to concomitant vaccination. Previous reviews and applied studies of China’s AEFI surveillance system have highlighted that passive surveillance data are susceptible to influences from reporting sensitivity, regional reporting practices, healthcare-seeking behavior, and event attribution, warranting cautious interpretation [16,18].

The significant interaction likely reflects heterogeneity in point estimates across doses rather than a consistent or clinically meaningful modification effect, as all dose-stratified confidence intervals were wide and crossed unity. Moreover, the direction of variation in AEFI reporting rates across doses was inconsistent, and trend analyses did not indicate a monotonic increase or decrease in AEFI incidence with increasing dose number. Given the small number of AEFI events at later doses, limited sample sizes for the third and fifth doses, and relatively wide confidence intervals for some stratified estimates, the observed dose-specific differences are more likely attributable to a combination of population characteristics, vaccination settings, and fluctuations in passive reporting rather than having clear clinical significance. Overall, no evidence was found to suggest a sustained increase in AEFI risk with successive sIPV doses, indicating good tolerability of multi-dose sIPV schedules. These findings are consistent with the overall safety profiles reported in previous studies of IPV or sIPV concomitant vaccination [16,24,29].

The independent association between younger age and higher AEFI reporting is consistent with established observations that post-vaccination reactions are more frequently reported in infants. This pattern likely reflects a combination of developmental differences in immune responsiveness during early life and greater sensitivity of caregivers and healthcare providers to symptoms occurring in younger children, consistent with previous studies showing higher AEFI reporting rates in younger age groups [18,30].

The strengths of this study include the use of large-scale real-world vaccination data, encompassing both standalone and multiple concomitant sIPV vaccination scenarios, thereby providing a comprehensive assessment of post-marketing safety. In addition, negative binomial regression models were applied to appropriately address overdispersion in low-incidence count data, and interaction terms and dose-stratified analyses were incorporated to enhance the robustness of risk estimation.

Several limitations should also be acknowledged. First, the AEFI surveillance system is a passive reporting system and may be subject to underreporting, incomplete information, and heterogeneity in reporting behavior. Second, the number of AEFI events for specific concomitant vaccination combinations and higher-dose administrations was limited, resulting in instability in some risk estimates. Future studies incorporating prospective active surveillance or multicenter designs are warranted to further validate the safety of concomitant sIPV vaccination. All subgroup and dose-specific analyses should be interpreted cautiously due to the low absolute number of AEFI events and the inherent limitations of passive surveillance data.

In summary, the findings indicate that sIPV, whether administered standalone or concomitantly with other routine vaccines, was not associated with a significant increase in AEFI reporting risk and that reported events were predominantly mild and transient general reactions. These results provide important real-world safety evidence supporting the continued use of sIPV in national immunization programs and concomitant vaccination strategies.

## 5. Conclusions

Based on large-scale real-world AEFI surveillance data from Anhui Province, the findings demonstrate that the Sabin strain inactivated poliovirus vaccine (sIPV) exhibits a favorable safety profile when administered standalone or concomitantly with other routine vaccines. The overall incidence of AEFI was low, with the vast majority of events being mild, self-limited general reactions, and serious adverse events were extremely rare. Concomitant vaccination did not significantly increase the risk of AEFI, and no evidence was observed for a sustained increase in risk with successive doses. These results support the safety of administering sIPV concomitantly with vaccines such as DTaP, Hib, and rotavirus within routine immunization programs and provide scientific evidence to inform optimization of immunization strategies and broader implementation of sIPV concomitant vaccination.

## Figures and Tables

**Table 1:** Demographic Baseline Characteristics (1 November 2023 to 31 July 2025).

Indicator	Standalone Vaccination (*N* = 135,550)	Concomitant Vaccination (*N* = 167,976)	Total (*N* = 303,526)	*p* Value
**Age (months)**				<0.0001
Mean (SD)	12.8 (24.00)	10.7 (19.90)	11.7 (21.85)	
Median (months)	3.0	3.0	3.0	
Range (Min–Max)	1–746	1–290	1–746	
**Age group**				<0.0001
<2 months, n (%)	20 (0.01)	10 (0.01)	30 (0.01)	
2 months, n (%)	64,494 (47.58)	36,794 (21.90)	101,288 (33.37)	
3 months, n (%)	27,700 (20.44)	66,741 (39.73)	94,441 (31.11)	
4–17 months, n (%)	15,537 (11.46)	35,749 (21.28)	51,286 (16.90)	
18 months–<4 years, n (%)	10,313 (7.61)	12,744 (7.59)	23,057 (7.60)	
≥4–<5 years, n (%)	8288 (6.11)	7478 (4.45)	15,766 (5.19)	
≥5–<9 years, n (%)	8841 (6.52)	8132 (4.84)	16,973 (5.59)	
≥9–<18 years, n (%)	335 (0.25)	324 (0.19)	659 (0.22)	
≥18 years, n (%)	22 (0.02)	4 (0.00)	26 (0.01)	
**Sex**				0.0056
Male, n (%)	71,955 (53.08)	90,015 (53.59)	161,970 (53.36)	
Female, n (%)	63,595 (46.92)	77,961 (46.41)	141,556 (46.64)	
**Region**				<0.0001
Fuyang City, n (%)	23,410 (17.27)	38,637 (23.00)	62,047 (20.44)	
Hefei City, n (%)	29,941 (22.09)	21,125 (12.58)	51,066 (16.82)	
Chuzhou City, n (%)	17,698 (13.06)	21,595 (12.86)	39,293 (12.95)	
Bozhou City, n (%)	7936 (5.85)	17,735 (10.56)	25,671 (8.46)	
Anqing City, n (%)	9873 (7.28)	12,589 (7.49)	22,462 (7.40)	
Lu’an City, n (%)	9886 (7.29)	8602 (5.12)	18,488 (6.09)	
Huainan City, n (%)	6349 (4.68)	7856 (4.68)	14,205 (4.68)	
Bengbu City, n (%)	4601 (3.39)	8071 (4.80)	12,672 (4.17)	
Huaibei City, n (%)	5331 (3.93)	5343 (3.18)	10,674 (3.52)	
Suzhou City, n (%)	6293 (4.64)	9677 (5.76)	15,970 (5.26)	
Other cities in Anhui Province, n (%)	12,831 (9.47)	14,607 (8.70)	27,438 (9.04)	
Other provinces, n (%)	1401 (1.03)	2139 (1.27)	3540 (1.17)	

**Table 2:** Overall Number of AEFI Cases and Incidence Rate (/100,000 doses).

Indicator	Standalone Vaccination (*N* = 135,550)	Concomitant Vaccination (*N* = 167,976)	Total (*N* = 303,526)	*p* Value
**Total AEFI**	79 (58.28)	109 (64.89)	188 (61.94)	0.4669
Time to onset				
Within 30 min	43 (31.72)	62 (36.91)	105 (34.59)	0.4449
0–3 days	78 (57.54)	107 (63.70)	185 (60.95)	0.4945
0–7 days	79 (58.28)	109 (64.89)	188 (61.94)	0.4669
0–30 days	79 (58.28)	109 (64.89)	188 (61.94)	0.4669
**AEFI type**				
General reaction	78 (57.54)	106 (63.10)	184 (60.62)	0.5361
Abnormal reaction	1 (0.74)	1 (0.60)	2 (0.66)	1.0000
Coincidental event	0 (0.00)	1 (0.60)	1 (0.33)	1.0000
Serious AEFI	0 (0.00)	2 (1.19)	2 (0.66)	0.5057
**Outcome**				
Improved	5 (3.69)	11 (6.55)	16 (5.27)	0.2806
Under treatment	0 (0.00)	1 (0.60)	1 (0.33)	1.0000
Recovered	74 (54.59)	97 (57.75)	171 (56.34)	0.7158

**Table 3:** Summary of AEFI Incidence Rates by Region.

Region	Cases	Proportion (%)	Doses	Rate (/100,000)	95% CI	Expected Cases	Std. Residual
Other cities in Anhui Province	10	5.32	27,438	36.45	17.45, 67.02	16.9947	−1.6967
Anqing	11	5.85	22,462	48.97	24.45, 87.61	13.9127	−0.7809
Bengbu	6	3.19	12,672	47.35	17.38, 103.03	7.8489	−0.6599
Chuzhou	5	2.66	39,293	12.73	4.13, 29.70	24.3376	−3.9198
Fuyang	101	53.72	62,047	162.78	132.61, 197.76	38.4311	10.0929
Hefei	15	7.98	51,066	29.37	16.44, 48.44	31.6296	−2.9569
Huaibei	6	3.19	10,674	56.21	20.63, 122.31	6.6113	−0.2378
Huainan	3	1.60	14,205	21.12	4.36, 61.71	8.7984	−1.9548
Lu’an	16	8.51	18,488	86.54	49.47, 140.50	11.4512	1.3442
Suzhou	5	2.66	15,970	31.31	10.17, 73.05	9.8916	−1.5553
Bozhou	10	5.32	25,671	38.95	18.68, 71.63	15.9003	−1.4797
Other Provinces	0	0	3540	0	-	2.1926	−1.4808

**Table 4:** Number of AEFI Cases and Incidence Rates (/100,000 doses) by Age, Sex, and Region.

Indicator	Standalone Vaccination *(N* = 135,550)	Concomitant Vaccination (*N* = 167,976)	Total (*N* = 303,526)	*p* Value
**Age**				
<2 months	0 (0.00)	2 (1.19)	2 (0.66)	0.5057
2 months	39 (28.77)	29 (17.26)	68 (22.40)	0.0352
3 months	20 (14.75)	34 (20.24)	54 (17.79)	0.2599
4–17 months	9 (6.64)	29 (17.26)	38 (12.52)	0.0093
18 months–<4 years	5 (3.69)	7 (4.17)	12 (3.95)	0.8349
≥4–<5 years	6 (4.43)	5 (2.98)	11 (3.62)	0.5556
≥5–<9 years	0 (0.00)	2 (1.19)	2 (0.66)	0.5057
≥9–<18 years	0 (0.00)	1 (0.60)	1 (0.33)	1.0000
**Sex**				
Male	42 (30.98)	64 (38.10)	106 (34.92)	0.2969
Female	37 (27.30)	45 (26.79)	82 (27.02)	0.9327
**Region**				
Fuyang	42 (30.98)	59 (35.12)	101 (33.28)	0.5342
Lu’an	5 (3.69)	11 (6.55)	16 (5.27)	0.2806
Hefei	10 (7.38)	5 (2.98)	15 (4.94)	0.0864
Anqing	5 (3.69)	6 (3.57)	11 (3.62)	1.0000
Other cities in Anhui Province	3 (2.21)	7 (4.17)	10 (3.29)	0.5277
Bozhou	2 (1.48)	8 (4.76)	10 (3.29)	0.2015
Bengbu	5 (3.69)	1 (0.60)	6 (1.98)	0.0956
Huaibei	0 (0.00)	6 (3.57)	6 (1.98)	0.0367
Chuzhou	4 (2.95)	1 (0.60)	5 (1.65)	0.1797
Suzhou	2 (1.48)	3 (1.79)	5 (1.65)	1.0000
Huainan	1 (0.74)	2 (1.19)	3 (0.99)	1.0000

**Table 5:** Number of AEFI Cases and Incidence Rates (/100,000 doses) by Dose Number.

Dose	Standalone Vaccination (*N* = 135,550)	Concomitant Vaccination (*N* = 167,976)	Total (*N* = 303,526)	Chi-Square Test, *p* Value	Stratified Negative Binomial Analysis, *p* Value	aRR Direction (Concomitant vs. Standalone)
Dose 1	48 (35.41)	38 (22.62)	86 (28.33)	0.0374	0.9543	1.01 (0.64–1.60)
Dose 2	19 (14.02)	55 (32.74)	74 (24.38)	0.0010	0.4400	0.78 (0.42–1.46)
Dose 3	2 (1.48)	3 (1.79)	5 (1.65)	1.0000	—	—
Dose 4	10 (7.38)	12 (7.14)	22 (7.25)	0.9401	0.6411	1.29 (0.44–3.81)
Dose 5	0 (0.00)	1 (0.60)	1 (0.33)	1.0000	—	—

**Table 6:** Number of Cases and Incidence Rates of Major AEFI Diagnoses (/100,000 doses).

Indicator	Standalone Vaccination (*N* = 135,550)	Concomitant Vaccination (*N* = 167,976)	Total (*N* = 303,526)	*p* Value
**AEFI (overall)**	79 (58.28)	109 (64.89)	188 (61.94)	0.4669
**General reactions**	78 (57.54)	106 (63.10)	184 (60.62)	0.5361
General reaction symptoms (fever/erythema/induration, etc.) *	75 (55.33)	103 (61.32)	178 (58.64)	0.4981
Fever	48 (35.40)	70 (41.68)	118 (38.88)	0.3831
37.1–37.5 °C	9 (6.64)	13 (7.74)	22 (7.25)	0.7228
37.6–38.5 °C	29 (21.39)	38 (22.63)	67 (22.07)	0.8196
≥38.6 °C	10 (7.38)	19 (11.31)	29 (9.55)	0.2699
Injection-site erythema/swelling	24 (17.70)	31 (18.46)	55 (18.12)	0.8776
≤2.5 cm	10 (7.38)	10 (5.95)	20 (6.59)	0.6315
2.6–5.0 cm	11 (8.11)	20 (11.91)	31 (10.21)	0.3036
>5.0 cm	3 (2.21)	1 (0.60)	4 (1.32)	0.3308
Injection-site induration	18 (13.28)	26 (15.48)	44 (14.50)	0.6159
≤2.5 cm	4 (2.95)	9 (5.36)	13 (4.28)	0.3134
2.6–5.0 cm	13 (9.59)	17 (10.12)	30 (9.88)	0.8830
>5.0 cm	1 (0.74)	0 (0.00)	1 (0.33)	0.4467
Allergic reaction –Rash allergic	3 (2.21)	3 (1.79)	6 (1.98)	1.0000
**Abnormal reactions**	1 (0.74)	1 (0.60)	2 (0.66)	1.0000
Allergic reaction –Rash allergic	1 (0.74)	0 (0.00)	1 (0.33)	0.4466
Thrombocytopenic purpura	0 (0.00)	1 (0.60)	1 (0.33)	1.0000
**Pending diagnosis**	0 (0.00)	1 (0.60)	1 (0.33)	1.0000
Thrombocytopenic purpura	0 (0.00)	1 (0.60)	1 (0.33)	1.0000
**Coincidental events**	0 (0.00)	1 (0.60)	1 (0.33)	1.0000
Upper respiratory tract infection viral	0 (0.00)	1 (0.60)	1 (0.33)	1.0000

Note: * If a single case presented with multiple symptoms (e.g., fever, erythema/swelling, and injection site induration), each symptom was counted as one event.

## Data Availability

The data supporting the findings of this study were obtained from the China National Adverse Events Following Immunization Surveillance System (CNAEFIS) and the Anhui Provincial Immunization Information Management System. Due to data protection regulations and ethical restrictions related to individual privacy, these data are not publicly available. Aggregated data supporting the conclusions of this study may be made available from the corresponding author upon reasonable request and with permission from the relevant authorities.

## Supplementary Materials

The following supporting information can be downloaded at: https://www.mdpi.com/article/10.3390/vaccines14020161/s1, Table S1: Distribution of vaccines co-administered with sIPV; Table S2: Distribution of AEFI for Vaccines Most Frequently Involved in Concomitant Administration with sIPV; Table S3: Clinical characteristics of reported thrombocytopenic purpura cases following vaccination; Table S4: Distribution of AEFI Cases and Incidence Rates by Age Group (/100,000 doses).

## Abbreviations

The following abbreviations are used in this manuscript:

sIPV	Sabin strain–based inactivated poliovirus vaccine
IPV	Inactivated poliovirus vaccine
OPV	Oral poliovirus vaccine
DTaP	Diphtheria–tetanus–acellular pertussis vaccine
DT	Diphtheria–tetanus vaccine
Hib	Haemophilus influenzae type b vaccine
MMR	Measles–mumps–rubella vaccine
BCG	Bacille Calmette–Guérin vaccine
EV71	Enterovirus 71 vaccine
VAPP	Vaccine-associated paralytic poliomyelitis
VDPV	Vaccine-derived poliovirus
AEFI	Adverse events following immunization
CNAEFIS	China National Adverse Events Following Immunization Surveillance System
WHO	World Health Organization
GPEI	Global Polio Eradication Initiative
RR	Relative risk
aRR	Adjusted rate ratio
CI	Confidence interval
NB	Negative binomial (regression)
NHC	National Health Commission of China
NMPA	National Medical Products Administration
CDC	Center for Disease Control and Prevention
