# Supplementary material for: Safety of Sabin Inactivated Poliovirus Vaccine Administered Standalone or Concomitantly with Other Childhood Vaccines: A Real-World Study in China
Source: Vaccines (Basel). 2026 Feb 9;14(2):161. doi: 10.3390/vaccines14020161 (PMC12945227; doi:10.3390/vaccines14020161)
Supplement: Supplementary file 1 [file vaccines-14-00161-s001.zip › vaccines-4116173-supplementary.pdf]

Table S1. Distribution of vaccines co-administered with sIPV.

Vaccine type	Number of doses	Proportion (%)
DTaP	117,878	58.97
Pentavalent rotavirus vaccine	19,525	9.77
Hib	13,583	6.79
DTaP–Hib quadrivalent vaccine	11,079	5.54
Hepatitis B	9,332	4.67
Varicella	6,872	3.44
DT	4,338	2.17
MMR	3,903	1.95
Meningococcal vaccine	3,680	1.84
Pneumococcal vaccine	2,152	1.08
Quadrivalent influenza vaccine	2,143	1.07
Rotavirus vaccine	1,925	0.96
Hepatitis A	1,546	0.77
Japanese encephalitis vaccine	1,384	0.69
BCG	165	0.08
EV71	135	0.07

Note: 1. Vaccines with relatively high proportions, including DTaP, pentavalent rotavirus vaccine, Hib vaccine, DTaP–Hib quadrivalent vaccine, and diphtheria–tetanus (DT) vaccine, were not combined. Other vaccines with lower usage were grouped according to valency, formulation (live attenuated/inactivated/lyophilized), and cell substrate. 2.Only vaccines accounting for more than 0.05% of the total were listed.

Table S2. Distribution of AEFI for Vaccines Most Frequently Involved in Concomitant Administration with sIPV

Concomitantly Administered Vaccine	Diagnosis	No. of AEFI Cases	Proportion of Total AEFI (%)
DTaP–Hib combination vaccine	General reactions (fever, erythema, induration, etc.)	6	5.50
	Thrombocytopenic purpura (suspected)	1	0.92
	Subtotal	7	6.42
DTaP	Allergic reaction – rash (pending diagnosis)	3	2.75
	Thrombocytopenic purpura	1	0.92
	General reactions (fever, erythema, induration, etc.)	75	68.81
	Coincidental event – viral upper respiratory infection	1	0.92
	Subtotal	80	73.39
Total AEFI associated with these concomitant vaccines	—	87	79.82

Percentages were calculated based on the total number of reported AEFI cases following concomitant administration during the study period.

Only vaccines accounting for the majority of reported AEFI following concomitant administration with sIPV are presented in this table.

Diagnoses were classified according to the National Surveillance Program for Suspected Adverse Events Following Immunization in China.

Table S3. Clinical characteristics of reported thrombocytopenic purpura cases following vaccination

Age (months)	Sex	Pre-vaccination Medical History	Vaccine 1 (Name & dose No.)	Vaccine 2 (Name & dose No.)	Date and Time of Onset	Clinical Course Description	Outcome	Final AEFI Classification	Final Causality Assessment
6.2	Male	None	Inactivated Poliovirus Vaccine (Sabin strain); 2nd dose	DTaP–Hib combined vaccine; 1st dose	14 July 2024, 15:00	According to the caregiver, a severe allergic reaction occurred in the afternoon on the day of vaccination, with petechial hemorrhages observed on the lower limbs. Three days later, the child was taken to the district people's hospital for blood testing, which revealed a platelet count of 7×10^9/L. The patient was subsequently transferred to a tertiary Hospital for treatment.	Improved	Abnormal reaction	Indeterminate (cannot be ruled out)
2.3	Male	None	Diphtheria–tetanus–acellular pertussis vaccine (DTaP); 1st dose	Inactivated Poliovirus Vaccine (Sabin strain); 1st dose	14 February 2025, 08:50	On 14 February 2025 at 08:22, the child received intramuscular injection of IPV in the right thigh and DTaP vaccine in the right upper arm. During the observation period, mild erythematous spots appeared on the face and body, and the caregiver was advised to monitor the condition. From 14 to 16 February, the rash gradually increased. On 16 February, the child was examined at the county people's hospital, where a platelet count of 13×10^9/L was detected, and the patient was transferred to a tertiary hospital for emergency treatment.	Improved	Under investigation	Not assessed

Table S4. Distribution of AEFI Cases and Incidence Rates by Age Group (/100,000 doses)

Age group	Indicator	Separate Vaccination (N = 135,550)	Concomitant Vaccination (N = 167,976)	Total (N = 303,526)	P value
**<2 months**	General reaction symptoms	0 (0.00)	2 (0.2)	2 (0.07)	0.1034
**2 months**	Allergic reaction – allergic rash	1 (1.55)	0 (0.00)	1 (0.99)	1.0000
	Thrombocytopenic purpura	0 (0.00)	1 (2.72)	1 (0.99)	0.3633
	General reaction symptoms	38 (58.92)	28 (76.10)	66 (65.16)	0.3028
**3 months**	Allergic reaction – allergic rash	1 (3.61)	2 (3.00)	3 (3.18)	1.0000
	General reaction symptoms	19 (68.59)	32 (47.95)	51 (54.00)	0.2137
**4–17 months**	Viral upper respiratory infection	0 (0.00)	1 (2.80)	1 (1.95)	1.0000
	Allergic reaction – allergic rash	2 (12.87)	1 (2.80)	3 (5.85)	0.2197
	Thrombocytopenic purpura	0 (0.00)	1 (2.80)	1 (1.95)	1.0000
	General reaction symptoms	7 (45.05)	26 (72.73)	33 (64.35)	0.2560
**18 months–<4 years**	General reaction symptoms	5 (48.48)	7 (54.93)	12 (52.04)	0.8310
**≥4–<5 years**	General reaction symptoms	6 (72.39)	5 (66.86)	11 (69.77)	0.8955
**≥5–<9 years**	General reaction symptoms	0 (0.00)	2 (24.59)	2 (11.78)	0.2295
**≥9–<18 years**	General reaction symptoms	0 (0.00)	1 (308.64)	1 (151.75)	0.4917

Comparisons involving sparse data should be interpreted cautiously.
